# Plaque‐adaptive flexible film dosimetry system for Ru‐106 plaque brachytherapy commissioning and quality assurance

**DOI:** 10.1002/mp.70286

**Published:** 2026-03-10

**Authors:** Jin Dong Cho, Hojae Kim, Dong Wook Kim, Heerim Nam, Jin Sung Kim, Jung‐in Kim

**Affiliations:** ^1^ Department of Radiation Oncology, Kangbuk Samsung Hospital Sungkyunkwan University School of Medicine Seoul Republic of Korea; ^2^ Department of Radiation Oncology, Yonsei Cancer Center, Heavy Ion Therapy Research Institute Yonsei University College of Medicine Seoul Republic of Korea; ^3^ Department of Radiation Oncology Yonsei University College of Medicine Seoul Republic of Korea; ^4^ Medical Physics and Biomedical Engineering Lab (MPBEL) Yonsei University College of Medicine Seoul Republic of Korea; ^5^ Oncosoft Seoul Republic of Korea; ^6^ Department of Radiation Oncology Seoul National University Hospital Seoul Republic of Korea; ^7^ Department of Radiation Oncology Seoul National University College of Medicine Seoul Republic of Korea; ^8^ Institute of Radiation Medicine Seoul National University Medical Research Center Seoul Republic of Korea; ^9^ Biomedical Research Institute Seoul National University Hospital Seoul Republic of Korea

**Keywords:** eye plaque brachytherapy, film dosimetry, quality assurance

## Abstract

**Background:**

Ruthenium‐106 (Ru‐106) plaque brachytherapy is an established treatment modality for choroidal melanoma that delivers localized beta radiation while sparing surrounding ocular structures. It is widely used in Europe and parts of Asia, whereas COMS Iodine‐125 and Palladium‐103 plaques remain the standard in the United States. However, its concave geometry presents challenges for accurate dose verification, as conventional flat dosimeters are ill‐suited for use during commissioning and quality assurance (QA).

**Purpose:**

This study aimed to assess the feasibility and dosimetric accuracy of a plaque‐adaptive flexible film dosimeter (PFD) for planar and central axis (CAX) dose measurements in Ru‐106 plaque brachytherapy.

**Methods:**

The PFD consisted of an active layer of lithium salt of pentacosa‐10,12‐diynoic acid (LiPCDA) enclosed between upper and lower silicone sheets. The flexible film was shaped to conform to the curvature of the Ru‐106 plaques and mounted within a dual‐layer film holder. A 3D‐printed QA tool was used for reproducible positioning. Dose measurements were performed at depths of 1 and 3 mm for CCA, COB, and CIB plaque types using a dual‐film configuration. Planar dose distributions were assessed at 33 manufacturer‐certified reference points at a 1 mm depth. CAX dose profiles were independently acquired using a microDiamond detector in a water phantom. All measurements were compared with manufacturer‐certified reference data. Calibration curves were established based on optical density and dose uncertainties were analyzed.

**Results:**

The dose ratio between 1 and 3 mm depths measured with the PFD differed by –0.2% to 0.4% from the reference data across all plaque types. Planar dose measurements at 33 points yielded a mean difference of 3.0%  ±  2.0% from reference values. In the high‐dose central region, the mean differences for CCA, COB, and CIB were 2.1%  ±  0.8%, 2.1%  ±  0.8%, and 2.2%  ±  0.7%, respectively. CAX dose profiles measured with a microDiamond detector differed by 0.89%  ±  0.58%. Total dose uncertainty ranged from 4.43% at 0.3 Gy to 4.19% at 2.4 Gy.

**Conclusions:**

The PFD enabled accurate and high‐resolution dose measurements on the curved surfaces of Ru‐106 plaques. Both planar and CAX measurements demonstrated strong agreement with manufacturer‐certified reference data across all plaque types. This system offers a practical and reproducible solution for commissioning and QA in Ru‐106 plaque brachytherapy, supporting its clinical integration for choroidal melanoma treatment.

## 1 INTRODUCTION

Eye plaque brachytherapy is a well‐established treatment modality for uveal melanoma, retinoblastoma, and other intraocular tumors. This technique involves the surgical placement of a radioactive plaque on the outer surface of the eye. It delivers localized radiation directly to the tumor while minimizing exposure to surrounding healthy tissues. It offers significant advantages, including a short treatment duration, ease of application, and preservation of vision, as it can often prevent the need for enucleation.^1^, ^2^, ^3^, ^4^, ^5^, ^6^

Among commonly used isotopes in eye plaque brachytherapy, Ruthenium‐106 (Ru‐106) is particularly effective due to its high‐energy beta emission—characterized by a maximum energy of 3.54 MeV and a mean of 1.42 MeV—and a half‐life of approximately 373.6 days, which enables a steep dose fall‐off, sparing critical ocular structures while maintaining effective tumor control.^7^, ^8^, ^9^, ^10^ Commercial Ru‐106 eye plaques are available in several configurations to accommodate different tumor locations and ocular geometries. Among these, the CCA, COB, and CIB eye plaque models (manufacturer‐designated model names; Eckert & Ziegler BEBIG, Berlin, Germany) are the most commonly used designs, corresponding to fully circular, optic‐notched, and iris‐notched geometries, respectively.^8^, ^11^, ^12^, ^13^, ^14^ Reliable and accurate dose delivery in eye plaque brachytherapy hinges on rigorous commissioning and quality assurance (QA) procedures, given the complexity of plaque geometry and the steep dose gradients involved. Task Group 129 of the American Association of Physicists in Medicine (AAPM) provides guidelines for dosimetry of COMS Iodine‐125 (I‐125) and Palladium‐103 (Pd‐103) eye plaques. In contrast, Task Group 221 offers comprehensive recommendations, including those specific to Ru106.^10^, ^15^ While these guidelines establish a robust framework, their practical implementation can be limited by challenges such as steep dose gradients, plaque curvature, and the lack of appropriate measurement tools for beta‐emitting sources. Although independent verification of Ru‐106 plaque dosimetry is technically demanding, studies have shown that this challenge can be addressed using various approaches, including EBT3 film‐based measurements, Monte Carlo simulations, and point detector methods with silica beads or diodes, often in conjunction with advanced phantoms, thereby enabling precise dose characterization under controlled experimental conditions.^14^, ^16^, ^17^ While these approaches have demonstrated promising accuracy, they often rely on flat dosimeters or setups that do not directly conform to the concave geometry of the eye plaque, limiting spatial resolution and reproducibility.

One of the key limitations in Ru‐106 plaque dosimetry arises from this intrinsic plaque curvature, which is designed to conform to the ocular surface. Conventional dosimetric tools such as flat radiochromic films and ionization chambers are not well suited for accurate measurement of spatial dose distribution and central axis (CAX) depth dose on concave surfaces.^10^

To address the limitations of conventional dosimeters in measuring dose distributions on concave surfaces, a flexible film dosimeter composed of lithium salts of pentacosa‐10,12‐diynoic acid (LiPCDA) was employed.^18^, ^19^ This material, which is structurally similar to Gafchromic EBT films, has demonstrated reliable performance in in vivo dosimetry for the skin, lens, and oral cavity, particularly in regions with anatomical irregularities.^18^, ^19^

In this study, the film was fabricated into a bi‐elliptical shape to match the inner curvature of Ru‐106 plaques, resulting in a plaque‐adaptive flexible film dosimeter (PFD). Similar kirigami‐ or origami‐inspired deformation strategies have been applied in stimuli‐responsive bilayer materials to enable flexible devices to conform to curved surfaces.^20^, ^21^ To facilitate depth‐specific measurements, a dual‐layer film holder made of acrylic was designed, allowing the simultaneous placement of two films at clinically relevant depths. A customized QA tool was also developed using three‐dimensional (3D) printing technology. This tool includes an eye plaque mounting base and a curved plunger, ensuring consistent and reproducible contact between the eye plaque and the film holder during measurement.

The proposed PFD system enables direct measurement of CAX and planar dose distributions on curved plaque surfaces. Its dosimetric accuracy and reproducibility were quantitatively validated through comparison with manufacturer‐certified reference data and independent measurements using a diamond ionization chamber. In current clinical practice, routine QA of Ru‐106 eye plaques primarily relies on manufacturer‐provided calibration data. These data include the CAX depth dose and two‐dimensional (2D) dose distributions measured in accordance with ISO 21439:2009(E). The manufacturer's data are typically used to verify source strength and dose uniformity. However, independent verification remains limited because no standardized dosimetric phantoms or detectors are available for β‐emitting sources. As a result, clinical physicists must rely on reference dose values provided by the manufacturer, which are usually reported with an expanded uncertainty of ±11% (*k* = 2). To address this limitation, the present study aimed to develop an independent verification system with an expanded uncertainty lower than ±11% by introducing a PFD capable of measuring depth‐ and profile‐specific doses with high spatial resolution. Furthermore, the PFD was validated as a clinical dosimetric tool to establish a plaque‐specific QA protocol for Ru‐106 brachytherapy, providing a practical and reliable approach for improving dosimetric accuracy and enhancing treatment safety in ocular applications.

## 2 METHODS

### 2.1 Preparation of plaque‐adaptive flexible film dosimeter

**FIGURE 1:**
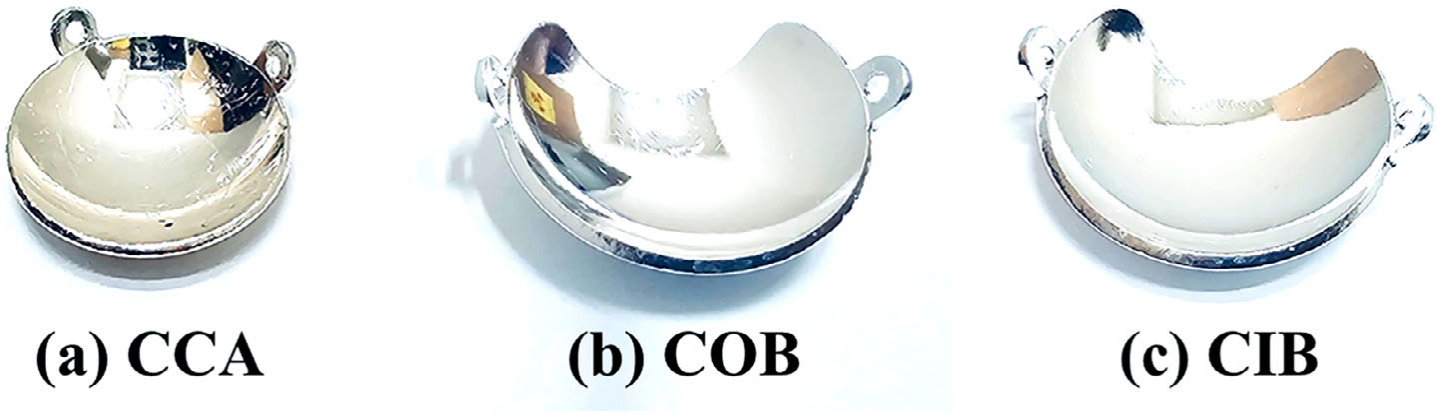
Representative photographs of Ru‐106 eye plaque models designated by the manufacturer (Eckert & Ziegler BEBIG GmbH, Berlin, Germany): (a) CCA, fully circular type; (b) COB, optic‐notched type; and (c) CIB, iris‐notched type. The concave surface of each plaque faces the tumor during treatment.

Commercial Ru‐106 plaques used in this study were obtained from Eckert & Ziegler BEBIG GmbH (Berlin, Germany). They are available in three main configurations—CCA, COB, and CIB—corresponding to fully circular, optic‐notched, and iris‐notched designs, respectively, as shown in Figure 1. A flexible film dosimeter was designed to conform to these plaque geometries for accurate dose verification and commissioning measurements.

The PFD used in this study was based on a radiochromic film composed of LiPCDA, which has been extensively characterized in previous studies on flexible in vivo and ocular dosimetry.^18^, ^19^, ^22^, ^23^ The active layer consists of LiPCDA microcrystals dispersed in a gelatin/polyvinyl‐alcohol matrix with a small amount of tartrazine to stabilize optical contrast.^24^, ^25^, ^26^, ^27^ This layer is enclosed between upper and lower silicone sheets, which provide mechanical flexibility, protect the active component from humidity and light, and ensure a water‐equivalent response during irradiation. The chemical structure and polymerization mechanism are identical to those of commercial Gafchromic EBT‐series films (EBT3 and EBT4), which also employ diacetylene‐based monomers. Therefore, the LiPCDA film is expected to exhibit similarly minimal intrinsic energy dependence between megavoltage photon and electron beams, consistent with previous characterization studies of Gafchromic EBT3 and EBT4 films in mixed photon–electron beam conditions.^28^, ^29^, ^30^, ^31^

Previous LiPCDA‐based dosimeters have demonstrated high spatial resolution, near‐tissue equivalence, and negligible dose‐rate dependence (100–1400 MU/min for 6–15 MV photons), with energy‐response variations below approximately 2% across megavoltage photon and electron beams.^19^, ^22^ The dose–response relationship follows a power law or low‐order polynomial function with excellent reproducibility (*R* ^2^ > 0.999). The optical signal remains stable when stored in the dark at room temperature (approximately 22°C) and scanned 24 h after irradiation.^18^, ^23^ Furthermore, cytotoxicity and irritation tests have confirmed the biocompatibility of the silicone‐encapsulated LiPCDA film, supporting its safe use for repeated clinical handling and ocular contact.^19^

For this study, a single dose–response calibration curve was generated for one production batch of LiPCDA film and applied to all measurements acquired during the same experimental period under controlled storage and scanning conditions. In clinical implementation, batch‐specific calibration and routine constancy checks of the film–scanner system are anticipated whenever Ru‐106 plaque QA is performed, consistent with established radiochromic film dosimetry protocols. Given the slow but measurable aging behavior reported for both LiPCDA and Gafchromic EBT‐series films, a full calibration should reasonably be repeated at least quarterly, or earlier if constancy checks reveal a systematic drift in the film–scanner response.^23^, ^32^ This calibration approach was consistently applied throughout the present work.

In this study, the same base LiPCDA formulation and calibration protocol were adopted. The film geometry and a dual‐layer acrylic holder were specifically designed to match the curvature and depth of Ru‐106 eye plaques for commissioning and QA measurements. The detailed fabrication and assembly procedures are described below.

The LiPCDA used in this work was synthesized in‐house from commercially available pentacosa‐10,12‐diynoic acid (PCDA, >98%, Alfa Aesar Korea Branch, Incheon, Korea) and lithium acetate (Guaranteed Reagent, Daejung Chemicals & Metals Co., Ltd., Korea). Synthesis was performed through controlled neutralization and polymerization steps following the procedure described in our previous studies.^18^, ^19^, ^22^, ^23^ Only the overall preparation route is summarized here, as the detailed chemical synthesis has been reported previously.

During synthesis, lithium acetate was used to neutralize PCDA and form the LiPCDA salt. This process yields microcrystalline diacetylene domains suitable for radiation‐induced topochemical polymerization in a gelatin/PVA matrix, consistent with previous structural and dosimetric investigations of LiPCDA‐based radiochromic materials.^22^, ^33^, ^34^, ^35^

The LiPCDA film was fabricated by casting the solution using an automatic coating machine and subsequently curing it at room temperature for 48 h. The final LiPCDA film had a thickness of approximately 150 ± 10 µm and was coated with silicone layers on both sides to protect the active layer and adjust the measurement depth. The upper and lower silicone layers were 250  ± 10 µm and 100 ± 10 µm thick, respectively. This asymmetric configuration was designed to align the effective measurement depths of the film with the reference depths used in manufacturer‐certified plaque dose data, thereby enabling accurate depth‐specific comparison. Before silicone coating, the thickness of each LiPCDA active layer was measured using a vernier caliper, and only films with uniform thickness were selected. The same quality control (QC) procedure was repeated after silicone coating to ensure dimensional consistency in the final PFD assembly.

**FIGURE 2:**
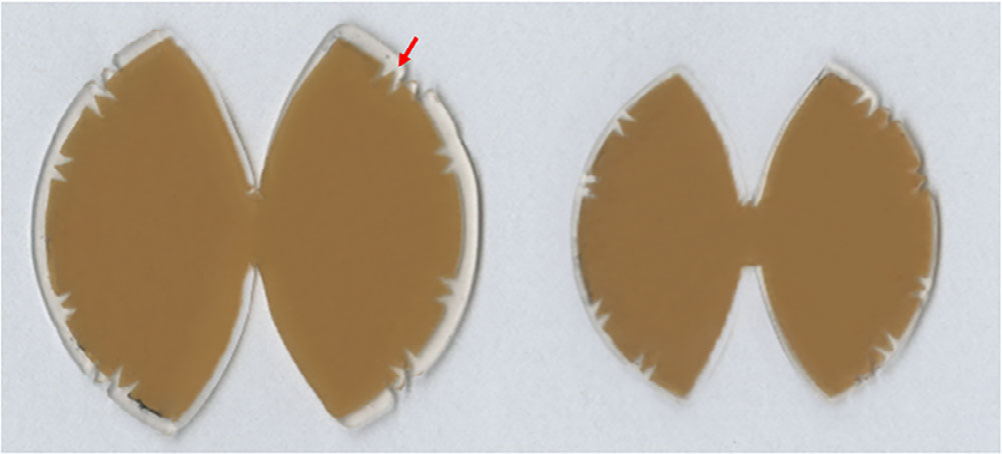
Plaque‐adaptive flexible film dosimeters (PFDs) fabricated by cutting flexible lithium salt of pentacosa‐10,12‐diynoic acid (LiPCDA) films into a bi‐elliptical shape. The red arrow indicates the reference marker, which is aligned along the *x*‐ and *y*‐axes, as well as both diagonal axes (d1 and d2). The PFD on the left is used in the first holder for dose measurements at a depth of 1 mm, and the PFD on the right is used in the second holder at a depth of 3 mm.

For placement within a curved eye plaque, the flexible silicone‐coated LiPCDA film was cut into a circular shape. To fit the circular plane LiPCDA film into the concave‐shaped eye plaque, the film was further trimmed into a bi‐elliptical shape using a cutting machine, the SILHOUETTE CAMEO 4 (Silhouette America, Inc., Lindon, UT, USA). To facilitate precise alignment after cutting the film into a bi‐elliptical shape, a triangular reference mark was inscribed at the center of the film. This mark extends symmetrically along the *x*‐ and *y*‐axes, as well as both diagonal directions (d1 and d2), as illustrated in Figure 2.

To enable dose measurements at varying depths within the eye plaque, a dual‐layer film holder was developed. Two PFDs were fabricated with diameters of 27.9 and 23.7 mm, respectively, to fit into the first and second holders of the assembly. These sizes were designed to ensure precise layered placement and film stability during measurement. The structural details and assembly process of the first and second holders are described in the following section.

### 2.2 Dual‐layer film holder assembly design

**FIGURE 3:**
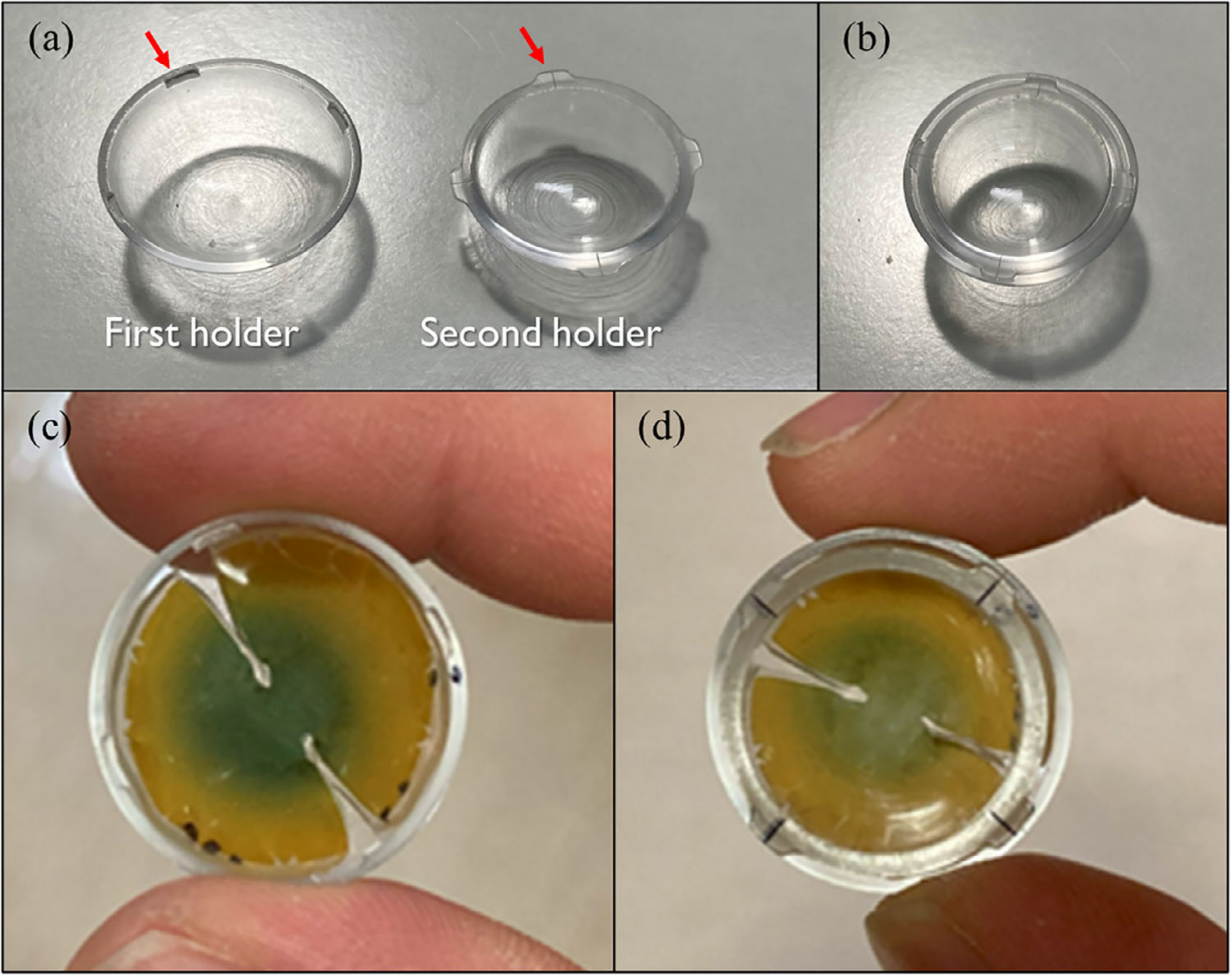
Assembly components and process for the dual‐layer film holder used in central axis (CAX) dose measurements. (a) First holder (left, 0.9 mm thick) and second holder (right, 1.5 mm thick), each fabricated from acrylic and equipped with interlocking protrusion‐recess features for precise alignment (red arrows). (b) Assembled state of the first and second holders. (c) Insertion of the first plaque‐adaptive flexible film dosimeters (PFD) (diameter: 27.9 mm) into the first holder, corresponding to a measurement depth of 1.0 mm from the inner surface of the eye plaque. (d) Final assembly with the second holder and second PFD (diameter: 23.7 mm) stacked above, enabling an additional measurement at 3.0 mm depth.

To measure the dose distribution and CAX dose of the Ru‐106 plaque, a dual‐layer film holder system was developed using custom‐fabricated acrylic holders. The components and assembly steps of the dual‐layer film holder are illustrated in Figure 3a,b. The dose distribution was evaluated at a depth of 1.0 mm from the concave inner surface of the plaque, while the CAX dose was measured at depths of 1.0 and 3.0 mm. These depths were achieved by sequentially stacking PFDs and acrylic holders of known thicknesses.

Two concentric holders were fabricated via computerized numerical control machining using acrylic, each with a thickness of 0.9 mm. The first PFD with a thickness of 0.5 mm was inserted between the first and second holders, allowing dose measurement at a depth of 1.0 mm. The second PFD with the same thickness was placed above the second holder to enable dose measurement at a depth of 3.0 mm from the plaque surface.

To ensure reproducible alignment and structural stability, interlocking features were integrated into both holders. These protrusion‐recess structures enabled precise positioning and secure fixation of the inserted films, minimizing movement during measurement. In addition, a reference scale was engraved on the second holder to ensure consistent alignment of pre‐marked films at a fixed angular position relative to the CAX of the plaque.

**FIGURE 4:**
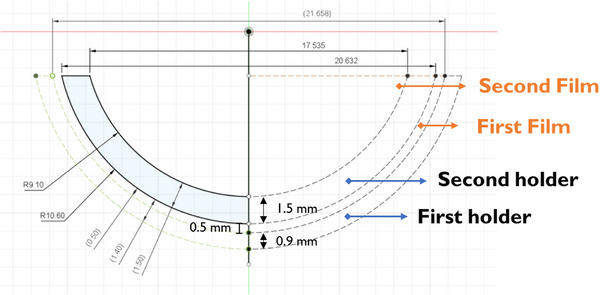
Cross‐sectional schematic of the dual‐layer film holder adapted to the curvature of Ru‐106 plaques (CCA, COB, and CIB types). The first and second films are positioned at depths of 1.0 and 3.0 mm from the inner surface of the plaque using two stacked acrylic holders, each 0.9 mm thick. Film thickness and interlayer spacing are indicated to illustrate depth‐specific placement relative to the concave plaque surface.

This dual‐layer configuration enabled precise and reproducible dose measurements at two distinct depths along the CAX on the curved surface of the eye plaque. The assembled configuration of the first and second holders with the inserted PFDs is shown in Figure 3c,d demonstrating the actual measurement setup. The geometric structure of the dual‐layer arrangement, including the relative film positions at 1.0 and 3.0 mm depths, is schematically illustrated in Figure 4.

To generate a film shape conforming to the hemispherical curvature of the Ru‐106 plaque, we first approximated the flattened contour of a hemispherical cap. This resulted in an asymmetric dome‐like shape resembling a distorted ellipse, where the upper and lower arc lengths differed due to the intrinsic geometric mismatch of flattening a non‐developable surface.^36^ Based on this geometry, a planar bi‐elliptical shape was constructed and iteratively trimmed while overlaying it onto the 3D curvature of the dual‐layer film holder. By removing peripheral mismatches, an optimized conformal profile was identified, minimizing wrinkling and detachment during plaque contact. This shaping process was guided by kirigami‐inspired principles, allowing improved conformity to the curved plaque surface.^20^, ^21^

### 2.3 Quality assurance tool design and assembly

To facilitate commissioning and QA measurements for Ru‐106 plaques, a customized QA tool was designed and fabricated using a 3D printer (MakerBot Replicator, MakerBot Industries, Brooklyn, NY, USA). The tool consists of two components: an eye plaque mounting base and a curved plunger.

The mounting base includes a cavity precisely shaped to fit the Ru‐106 plaque, ensuring stable positioning during measurements. A hemispherical or cubic recess was designed at the center of the base to accommodate the head of the curved plunger. The plunger itself has a hemispherical tip that conforms to the curvature of the dual‐layer film holder, allowing uniform pressure to be applied to the films and holder during assembly and irradiation.

**FIGURE 5:**
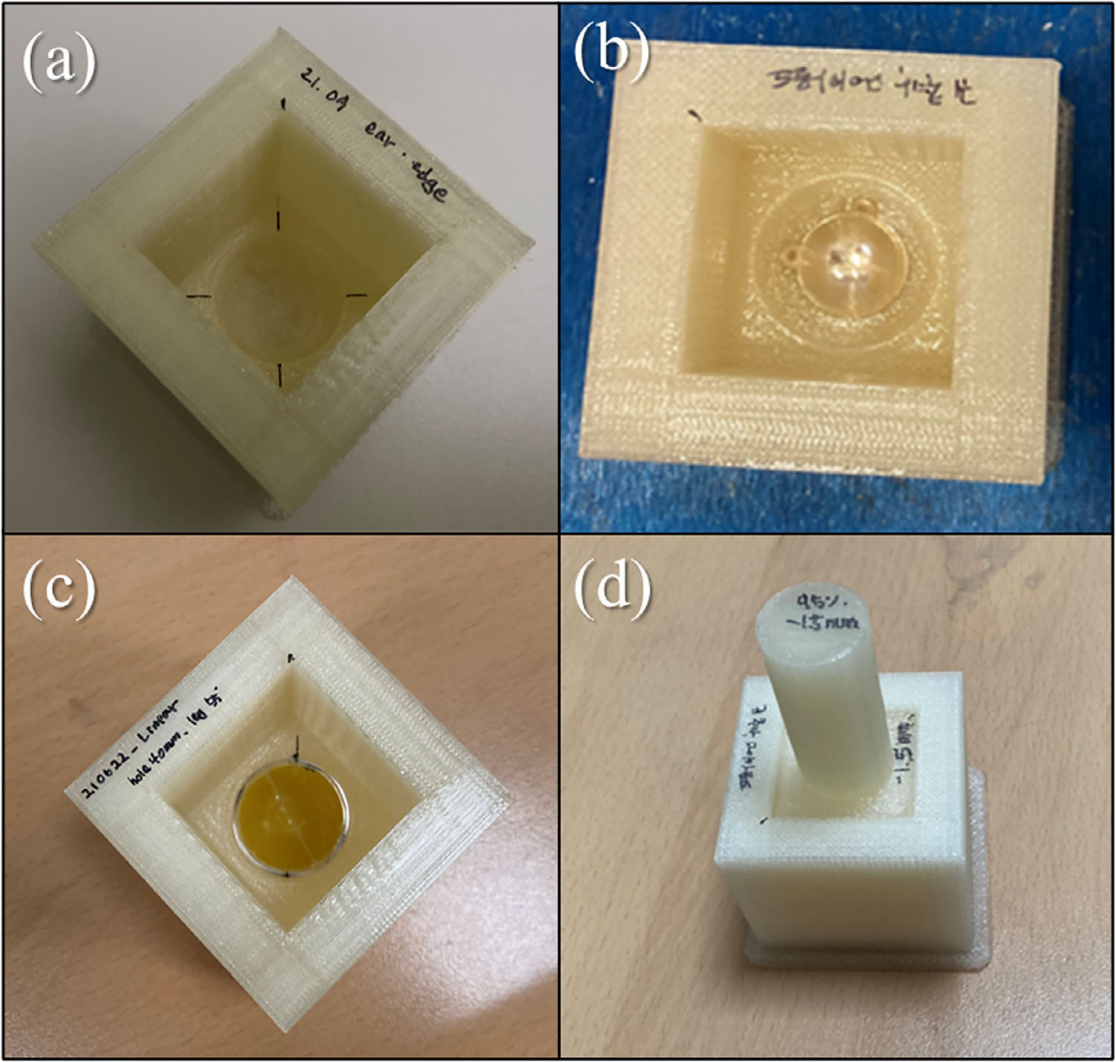
Photographs of the custom 3D‐printed QA tool designed for reproducible dosimetry with Ru‐106 plaques. (a) Base unit with a recessed area for plaque placement and alignment markers. (b) Eye plaque positioned within the base component for dosimetric measurement. (c) Dual‐layer film holder containing bi‐elliptical lithium salt of pentacosa‐10,12‐diynoic acid (LiPCDA) films placed on top of the mounted plaque. (d) Curved plunger assembled with the mounting base to ensure uniform contact between the plaque and film holder during irradiation.

The QA tool was printed using polylactic acid (PLA) with a fill density exceeding 95%. The printed structure was evaluated using CT imaging. The measured Hounsfield Unit (HU) values were converted to physical density through an image‐value‐to‐density table (IVDT) calibration. The final printed piece exhibited a density of approximately 1.1 g/cm^3^, confirming its near water equivalence. To maintain electronic equilibrium and ensure accurate dosimetric conditions, the tool was designed with a 2 cm‐thick surrounding wall, providing sufficient scatter material in all directions. The physical structure and assembly of the QA tool, including the eye plaque mounting base and curved plunger, are illustrated in Figure 5.

In our institution, Ru‐106 plaques are procured annually because of the approximately 1‐year half‐life of the isotope. Upon receipt and verification of newly supplied plaques, QA measurements are performed immediately. The manufacturer also provides dummy plaques consisting solely of a silver substrate without radioactive material; therefore, all QA procedures are conducted using the actual clinical plaques. After completion of QA, the plaques are cleaned with deionized water, ultrasonically washed, and stored in a radiation‐shielded facility. Before clinical use, the plaques undergo gas sterilization in accordance with institutional radiation safety and infection control protocols.

### 2.4 Measurement setup and scanning protocol

#### 2.4.1 microDiamond detector measurements

The CAX dose of the Ru‐106 plaque was measured using a PTW 60019 microDiamond detector integrated with an automated MP1 water phantom system (PTW, Freiburg, Germany) capable of precise vertical movement. The CCA model of the eye plaque was selected for these measurements. A custom‐designed mounting fixture, fabricated using 3D printing, was placed at the bottom center of the water phantom to secure the CCA plaque in position. The CAX of the detector, the plaque, and the fixture were carefully aligned to ensure accurate dose acquisition.

The microDiamond detector was positioned vertically and aligned along the CAX of the eye plaque. When the detector tip made contact with the plaque surface, the curved geometry of the plaque resulted in an air gap of approximately 0.5 mm along the CAX. In addition, the sensitive volume of the detector is located approximately 1.0 mm from the detector tip.^37^ Taking both the air gap and detector geometry into account, dose measurements were performed from 1.5 to 10.5 mm in 1 mm increments.

The manufacturer‐certified reference data included reference dose values at eleven discrete depths ranging from 0.83 to 10.00 mm, derived from a quartic polynomial fit. To enable direct comparison, the measured values were fitted using a quartic polynomial function, and fitted dose values were calculated at the same 11 certified depths. Each depth was measured for 1 min.

#### 2.4.2 PFD dose measurement and analysis

Dose measurements and calibration were performed using the previously described dual‐layer film holder and customized QA tool setup. The CCA plaque was positioned in the mounting base, and irradiations were performed at durations of 2.5, 5, 10, 15, and 20 min using a dose rate of 120.1 mGy/min. This value was derived by applying radioactive decay correction to the certified reference dose rate on the date of the experiment. The delivered dose (D) was determined by multiplying the dose rate with the irradiation time.

A dose–response curve was established by measuring the net optical density (netOD) of the first PFD as a function of the delivered dose. All irradiated films were pre‐scanned and rescanned 24 h after irradiation using an Epson 10000XL flatbed scanner in reflection mode at 300 dpi. Each film was centered on the scan bed and aligned along the scanner's longitudinal axis to ensure consistent orientation between scans. Image analysis was performed using ImageJ software (version 1.52a, US National Institutes of Health, Bethesda, MD, USA). Only the red channel was used for image analysis due to its superior dose sensitivity. Pixel values (PVs) were extracted from a central region of interest (ROI) and converted into netOD values using the following equation:

(1) $$\mathrm{netOD}=\mathrm{OD}-OD_{0}=\mathrm{lo}g_{10}\left(\frac{P-P_{\mathrm{bg}}}{P_{0}-P_{\mathrm{bg}}}\right)$$

where P is the post‐irradiation PV, $P_{0}$ is the pre‐irradiation PV, and $P_{\mathrm{bg}}$ is the background signal.

The netOD was normalized to zero at 0 Gy using:

(2) $$\Delta \mathrm{netOD}=\mathrm{netOD}-\mathrm{netO}D_{0}$$

The relationship between dose and ΔnetOD was modeled using a power‐law function:

(3) $$\Delta \mathrm{netOD}=aD+bD^{n}$$

(4) $$D=a\cdot \Delta n\mathrm{etOD}+b\cdot \Delta \mathrm{netO}D^{n}$$

where a, b, and n are fitting parameters. This inverse function was used to convert measured netOD values to absolute dose for subsequent analysis.

To quantify measurement precision, three independent films were irradiated for 15 min, and the resulting data were used to calculate the mean and standard deviation. Spatial dose distributions were obtained from the first PFD by aligning circular regions of interest (ROIs) with a 1 mm diameter to 33 reference points specified in the manufacturer certification. The ROIs were arranged along the *x*‐, *y*‐, and diagonal axes, centered around the triangular cutout of the PFD for consistent alignment.

**FIGURE 6:**
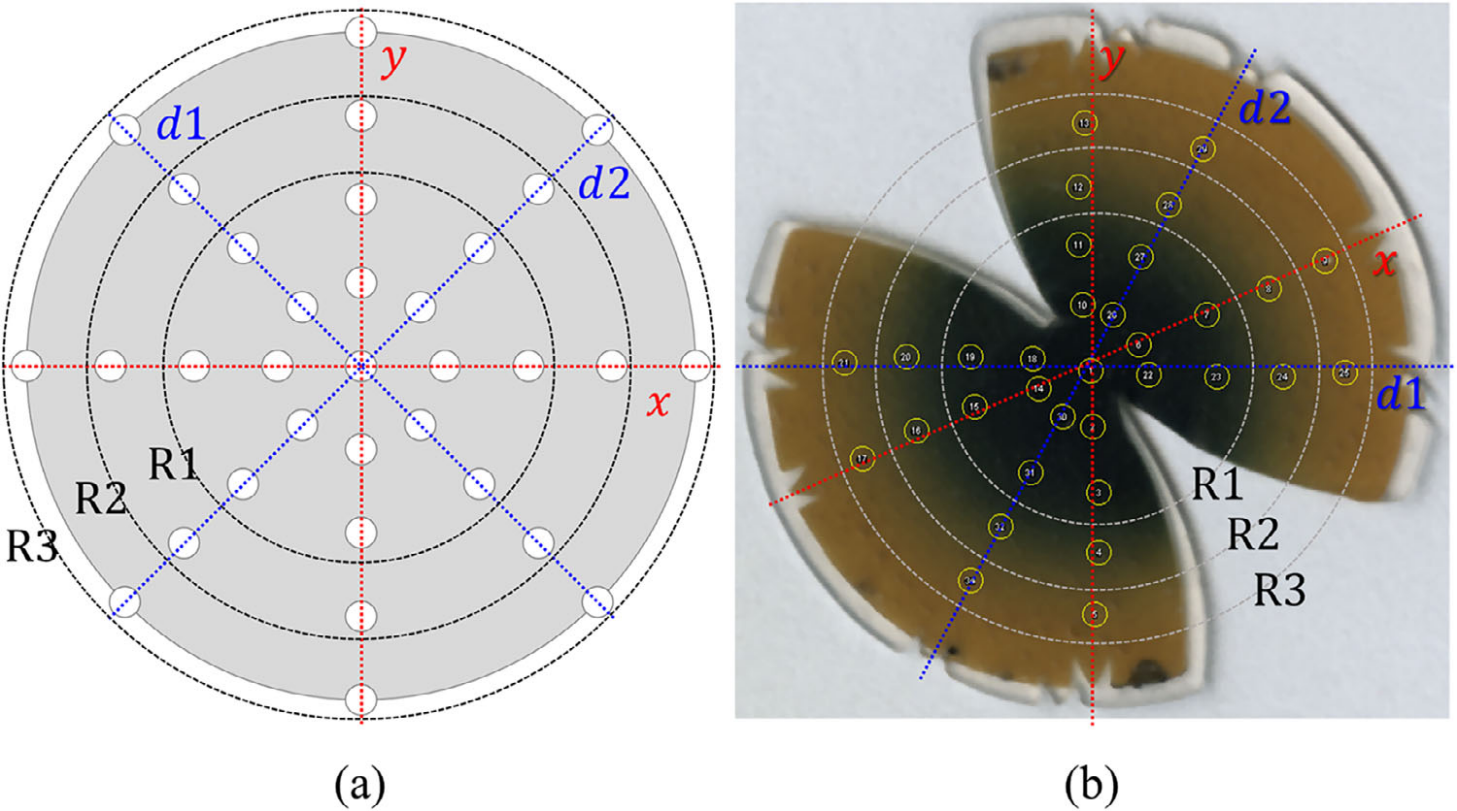
Reference map and scanned film image illustrating dose measurement locations for planar dosimetry. (a) Certified dose points (white circles) arranged along *x*‐, *y*‐, and diagonal (d1, d2) axes, grouped into radial zones R1–R3. (b) Scanned image of an irradiated film showing selected measurement points (yellow circles) aligned with reference positions. Reference notches define the *x*‐, *y*‐, and diagonal (d_1_, d_2_) axes for consistent alignment within the plaque holder.

A total of 33 points were identified on each film, with inter‐point distances ranging from 2.55 to 3.37 mm, depending on the eye plaque model. The diameters of the plaques were 15.3 mm for the CCA type, 19.8 mm for the COB type, and 20.2 mm for the CIB type. As shown in Figure 6, the central region R1 contained 17 measurement points, the intermediate region R2 contained 25 points, and the outermost region R3 encompassed all 33 points.

Points receiving less than 5% of the maximum dose were excluded from analysis. In the COB and CIB plaques, several peripheral points in the R3 region were omitted because the dose in those regions fell below the 5% threshold due to the low‐dose nature of the outermost areas and plaque geometry.

Planar dose distributions for CCA, COB, and CIB plaques were measured using the first PFD irradiated for 15 min. NetOD from each ROI was converted into dose using the established calibration curve, and the resulting spatial distributions were compared with manufacturer‐certified reference data.

CAX dose measurements at 1 and 3 mm depths were obtained from the first and second PFDs, respectively, following 15‐min irradiation. The CAX dose ratio was calculated and compared with manufacturer‐certified reference data.

To estimate the dose uncertainty associated with the bi‐elliptical LiPCDA film dosimetry, an error propagation method was applied based on the formalism proposed by Devic et al. and expanded in subsequent formulations for film‐based dose uncertainty analysis.^18^, ^19^, ^23^, ^38^, ^39^ The analysis incorporated both experimental and calibration fitting components, focusing on the relationship between PVs, netOD, and dose.

The standard deviation (SD) of netOD was calculated using the following propagation formula:

(5) $$\mathrm{SD}\left(\mathrm{netOD}\right)=\frac{1}{\ln10}\;\sqrt{\frac{\mathrm{SD}{\left(P_{0}\right)}^{2}}{{\left(P_{0}-P_{\mathrm{bg}}\right)}^{2}}+\frac{\mathrm{SD}{\left(P\right)}^{2}}{{\left(P-P_{\mathrm{bg}}\right)}^{2}}}$$

where $\mathrm{SD}(P_{0})$ and SD(P) represented the SDs of $P_{0}$ and P, respectively.

The total dose uncertainty ($SD_{\mathrm{tot}}$) was calculated as the quadratic sum of the experimental and fitting components:

(6) $$SD_{\mathrm{tot}}\left(\%\right)=\sqrt{{\mathrm{SD}}_{\exp}^{2}\left(\%\right)+SD_{\mathrm{fit}}^{2}\left(\%\right)}$$

The fitting uncertainty ($SD_{\mathrm{fit}}$) was computed based on the SDs of the fitting parameters:

(7) $$SD_{\mathrm{fit}}\left(\%\right)=\frac{\sqrt{\mathrm{net}\;OD^{2}\cdot {\mathrm{SD}}_{a}^{2}+\mathrm{netO}D^{2n}\cdot {\mathrm{SD}}_{b}^{2}}}{D_{\mathrm{fit}}}\times 100$$

The experimental uncertainty ($SD_{\exp}$) was derived from the following expression:

(8) $$SD_{\exp}\left(\%\right)=\frac{\left(a+n\cdot b\cdot \mathrm{net}\;OD^{n-1}\right)\cdot \mathrm{SD}\left(\mathrm{net}\;\mathrm{OD}\right)}{D_{\mathrm{fit}}}\times 100$$

here, *a*, *b*, and *n* are parameters derived from Equation (4), and $SD_{a}$ and $SD_{b}$ are the SDs of a and b, respectively.

## 3 RESULTS

### 3.1 Scanning properties

**FIGURE 7:**
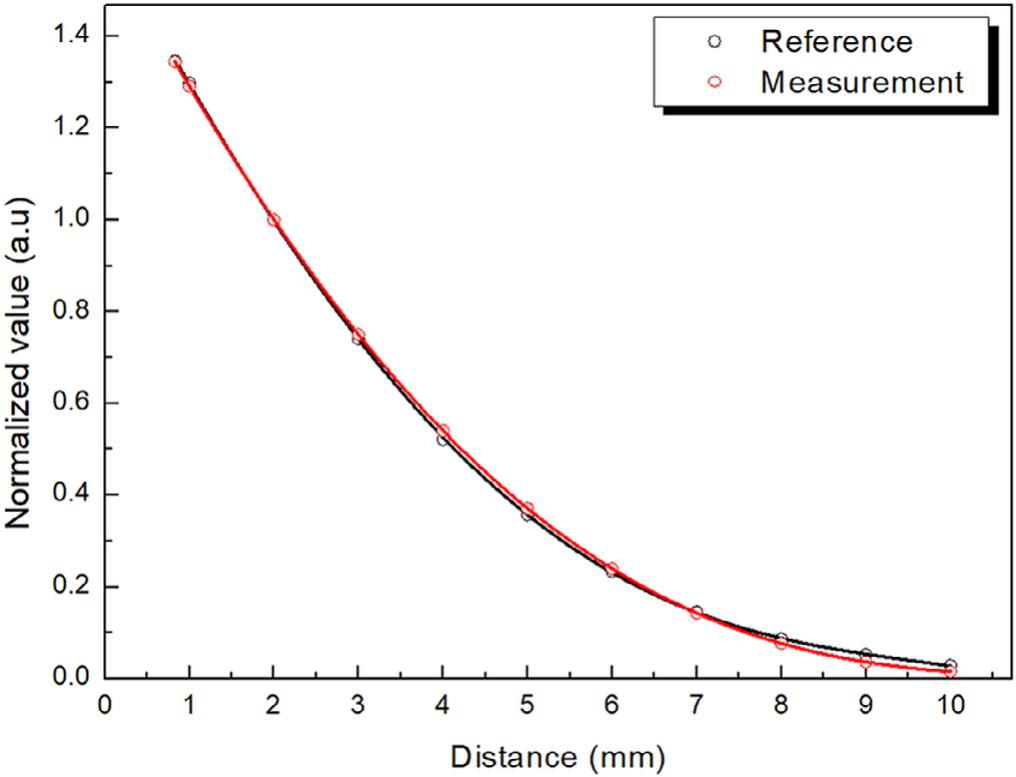
Central axis (CAX) dose profile of the CCA eye plaque measured using a microDiamond detector. Measured values were normalized at a depth of 2 mm and compared with reference data provided by the manufacturer.

The CAX dose profile of the CCA eye plaque was measured using a microDiamond detector in a water phantom, as shown in Figure 7. All values were normalized to the dose at a depth of 2 mm and compared with manufacturer‐certified reference data. Table 1 presents the normalized dose values and corresponding percentage deviations at 11 depths ranging from 0.83 to 10.00 mm. The mean deviation from the reference profile was 0.89%, with a standard deviation of 0.58%. The largest negative deviation was −1.57% at 9 mm depth. All measured values remained within ±2% of the reference data, confirming the accuracy of the microDiamond detector and the reliability of the measurement setup for CAX dose verification in Ru‐106 plaque dosimetry.

**TABLE 1:** Depth dose values along the central axis (CAX) of the CCA plaque, measured using a microDiamond detector.

Distance (mm)	Reference	Measurement	Difference (%)
0.83	1.35	1.34	−0.16
1.00	1.30	1.29	−0.70
2.00	1.00	1.00	0.00
3.00	0.74	0.75	0.93
4.00	0.52	0.54	1.93
5.00	0.36	0.37	1.34
6.00	0.23	0.24	0.62
7.00	0.15	0.14	−0.28
8.00	0.09	0.08	−0.94
9.00	0.05	0.04	−1.57
10.00	0.03	0.02	−1.31

*Note*: All values are normalized at a depth of 2 mm and compared with manufacturer‐certified reference data.

### 3.2 Dosimetric properties

**FIGURE 8:**
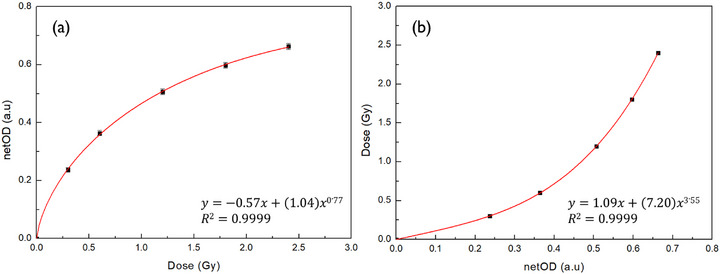
(a) Dose–response curve showing net optical density (netOD) as a function of delivered dose for PFD up to 2.4 Gy. The data were fitted using a power‐law model (Equation 3). (b) Calibration curve expressing dose as a function of netOD, used for converting measured optical densities to dose (Equation 4).

The dose–response relationship was derived from the first PFD irradiated at a depth of 1 mm using the Ru‐106 CCA plaque, as described in the calibration procedure. As shown in Figure 8a, the relationship between the delivered dose and netOD was modeled using a power‐law function up to 2.4 Gy. The inverse of this function, plotted in Figure 8b, was used to construct a calibration curve for dose conversion. The coefficient of determination (*R* ^2^) exceeded 0.9999 for both models, indicating excellent agreement between the fitted curve and the measured data.

**FIGURE 9:**
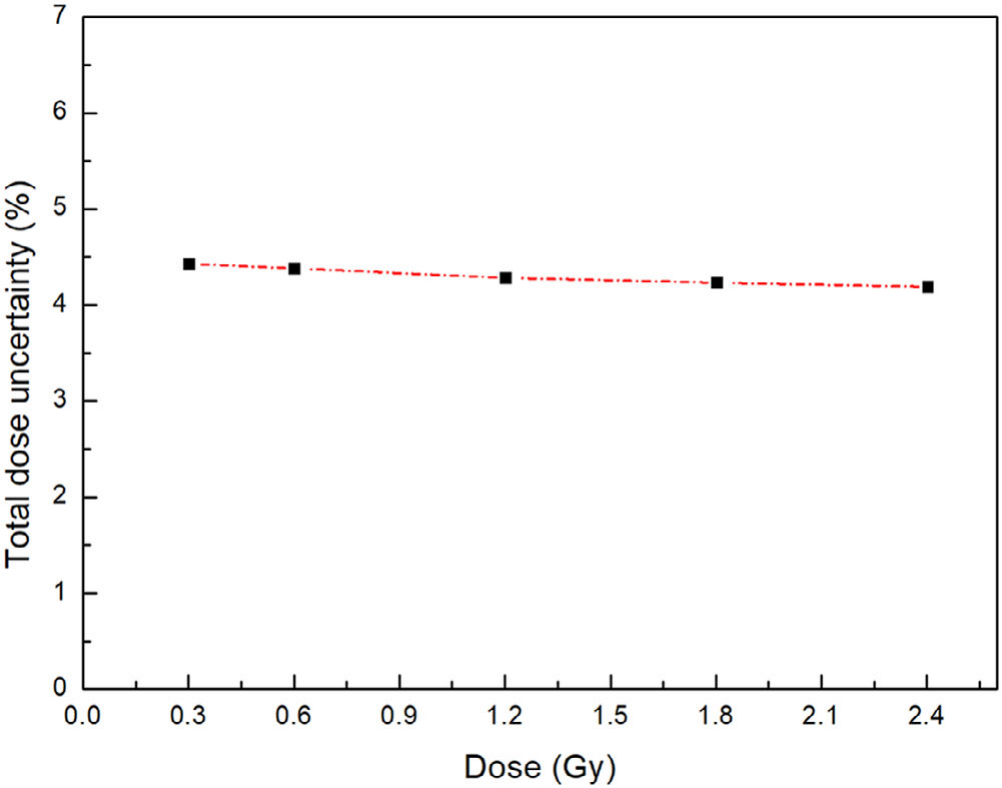
Total dose uncertainty of the plaque‐adaptive flexible film dosimeter (PFD) as a function of delivered dose. The uncertainty was calculated by combining experimental and fitting components using an error propagation method.

Total dose uncertainties were evaluated across the full dose range using an error propagation method incorporating both experimental and curve‐fitting components. As shown in Figure 9, the total dose uncertainty ranged from 4.43% at 0.3 Gy to 4.19% at 2.4 Gy within the investigated dose range. As no data were acquired below 0.3 Gy, this low‐dose region was not included in the validated operational range. Due to expected signal degradation and increased uncertainty in this range, no extrapolation was performed.^18^, ^19^, ^40^, ^41^ The individual uncertainty values corresponding to each dose point are summarized in Table 2.

**TABLE 2:** Individual total dose uncertainty values corresponding to Figure 9.

Dose (Gy)	Total dose uncertainty (%)
0.30	4.43
0.60	4.38
1.20	4.28
1.80	4.23
2.40	4.19

### 3.3 Central axis dose ratio and planar dosimetry

The CAX dose ratio is a key dosimetric parameter in Ru‐106 plaque brachytherapy, reflecting the steep depth‐dose gradient of beta‐emitting sources. Accurate verification of the dose ratio between 1 and 3 mm depths is essential to ensure safe and effective dose delivery to ocular tumors while sparing healthy tissues.^13^, ^42^, ^43^

To evaluate the accuracy of the PFD system, doses were measured at 1 and 3 mm depths for each plaque type (CCA, COB, CIB), and the dose ratio between first and second PFD was compared with manufacturer‐certified reference data. Table 3 summarizes the measured doses, the measured‐to‐reference ratios, and the certification values.

**TABLE 3:** Central axis (CAX) dose at 1 and 3 mm depths using plaque‐adaptive flexible film dosimeter for CCA, COB, and CIB plaques.

	Film and depth	Dose (Gy)	Ratio first to second	Reference	Difference
CCA	First film (1 mm)	179.5 ± 3.2	57.4%	57.0%	0.4%
	Second film (3 mm)	103.0 ± 2.3			
COB	First film (1 mm)	157.4 ± 1.1	57.9%	58.1%	−0.2%
	Second film (3 mm)	91.2 ± 1.4			
CIB	First film (1 mm)	201.7 ± 2.3	55.8%	55.3%	0.4%
	Second film (3 mm)	112.5 ± 2.0			

*Note*: Dose ratios between the two depths were calculated and compared with certified reference values.

The differences between measured and certified dose ratios ranged from −0.2% to 0.4%, indicating strong agreement across all plaque types. These findings demonstrate that the dual‐layer film holder and the PFD system provide precise and reproducible CAX dose measurements.

The consistency of the results also highlights the mechanical stability and repeatability of the customized QA setup, supporting its suitability for routine clinical use in plaque commissioning and QA.

**FIGURE 10:**
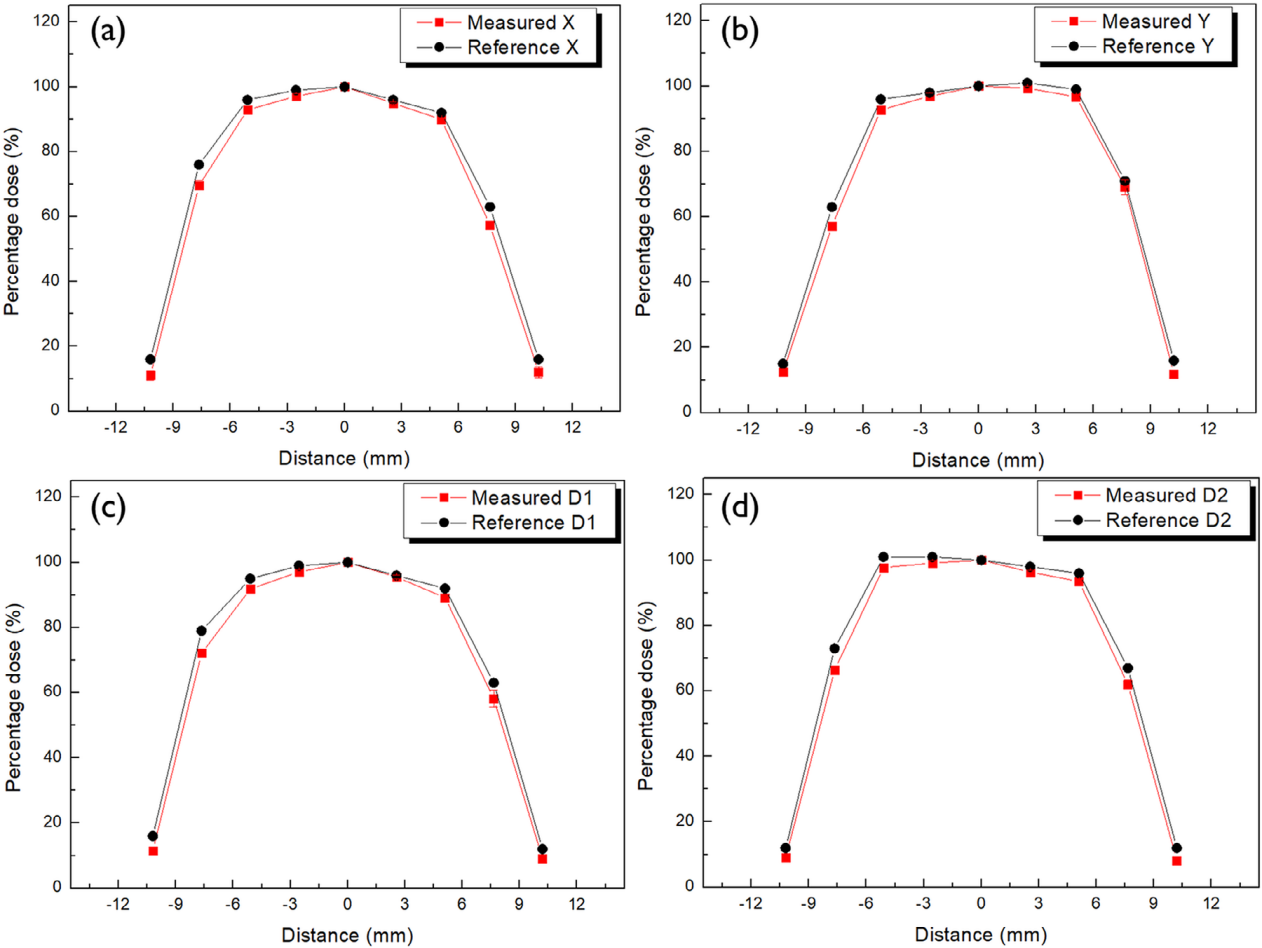
Planar dose distribution of the CCA plaque measured at 1 mm depth using plaque‐adaptive flexible film dosimeter (PFD). Measured dose profiles (red) are compared with manufacturer‐certified reference data (black) along the *x*‐, *y*‐, and diagonal (d1 and d2) axes.

**FIGURE 11:**
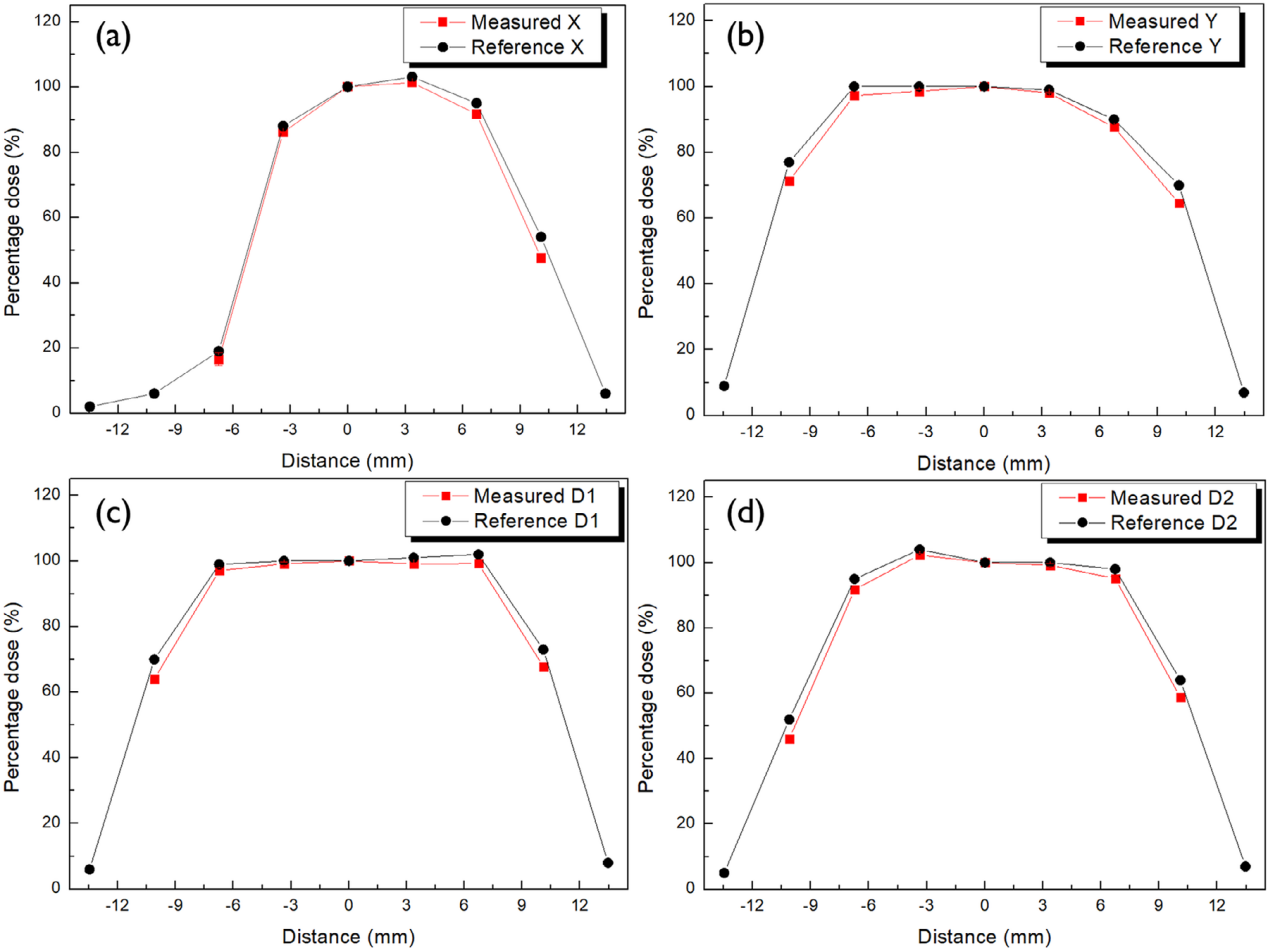
Planar dose profile of the COB eye plaque at 1 mm depth, showing dose distribution agreement between lithium salt of pentacosa‐10,12‐diynoic acid (LiPCDA) film measurements and manufacturer certification.

**FIGURE 12:**
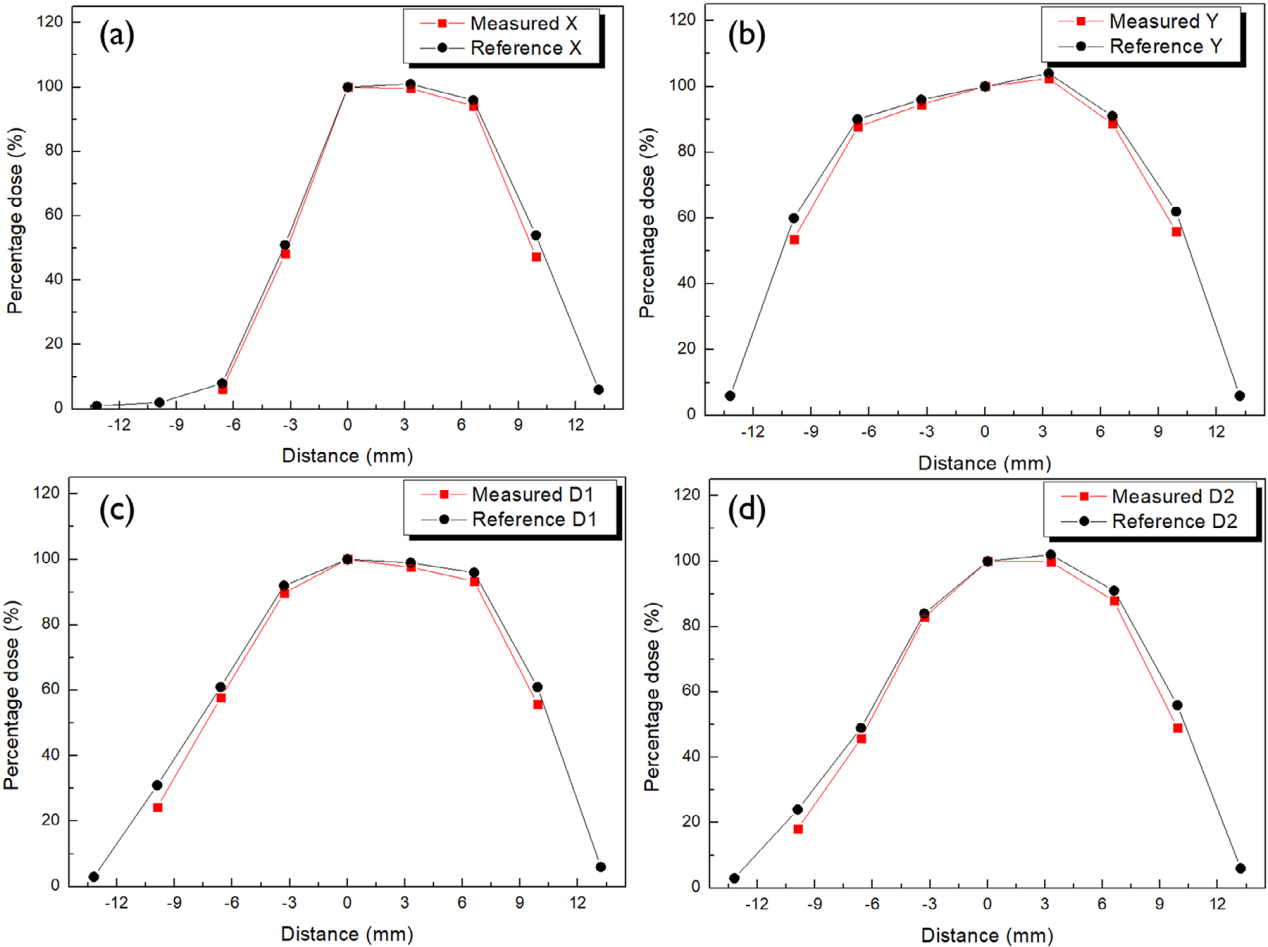
Planar dose profile of the CIB eye plaque at 1 mm depth, with dose measurements along key radial axes compared against certified values.

To evaluate the planar dose distribution of Ru‐106 plaques, PFDs were used to obtain dose profiles along the *x*‐, *y*‐, and diagonal (d1 and d2) axes at a depth of 1 mm from the plaque surface for each model—CCA, COB, and CIB. Figures 10, 11, 12 display the measured dose distributions derived from the profiles along the *x*‐, *y*‐, and diagonal (d1 and d2) axes, directly compared with the reference data provided by the manufacturer. The measured distributions, shown in red, exhibited strong agreement with the reference values in black, validating the applicability of the PFD system to concave geometries.

Quantitative comparisons between measured and reference doses were conducted across three radial regions—R1, R2, and R3—with results summarized in Table 4 for each plaque model (CCA, COB, and CIB). All deviations remained within approximately 3%, indicating a high level of dosimetric agreement.

**TABLE 4:** Mean dose differences between measurements and reference values for each plaque type.

Type	Radial point count (*n*)	Mean ± SD (%)
CCA	17 (R1)	2.1 ± 0.8
	25 (R2)	3.2 ± 1.9
	33 (R3)	3.3 ± 1.7
COB	17 (R1)	2.1 ± 0.8
	25 (R2)	3.1 ± 1.8
CIB	17 (R1)	2.2 ± 0.7
	24 (R2)	3.3 ± 2.1

*Note*: Values are summarized by radial region: R1 (innermost), R2 (intermediate), and R3 (outermost).

The R1 region consistently exhibited the smallest discrepancies, reflecting its relatively uniform dose distribution and clinical importance. In contrast, larger differences observed in the R2 and R3 regions, particularly in the COB and CIB plaques, are attributed to steep dose gradients near the plaque periphery, where minor spatial uncertainties can amplify dosimetric variation.

Overall, the flexible film dosimetry system demonstrated accurate and reproducible performance across concave geometries. Its strong agreement with certified reference data, especially in regions critical for tumor control, underscores its value in plaque commissioning and routine QA.

## 4 DISCUSSION

In this study, a PFD system was developed and validated for Ru‐106 plaque brachytherapy. The system enabled accurate 11% measurements of CAX dose ratios at 1 and 3 mm depths, as well as dose distributions on curved plaque surfaces.

The measured CAX dose ratios were within ±0.4% of the certified values across all plaque types, including CCA, COB, and CIB, demonstrating the high accuracy and reproducibility of the dual‐layer film holder setup. This agreement underscores the suitability of the system for routine verification of the steep dose gradients characteristic of beta‐emitting Ru‐106 plaques.

Planar dose evaluation revealed that mean dose differences remained within 3.3% in clinically relevant regions, including the R1 and R3 regions, with slightly larger deviations observed in peripheral areas, particularly for the COB and CIB plaques. These peripheral discrepancies likely stem from steep dose gradients and minor spatial uncertainties, consistent with geometric dose fall‐off near plaque edges. The R3 region was analyzed only for CCA due to low signal (<5% of max dose) in other plaque types, where lower signal‐to‐noise ratios further contribute to measurement uncertainty.

The slightly steeper falloff and narrower lateral dose profiles observed in the film measurements can be attributed to geometric differences between the flattened film and the curved plaque geometry. In the reference data, doses were acquired on the concave plaque surface and expressed along the chord length. In contrast, the film—although irradiated in a conformal shape—was scanned in a planar configuration, effectively mapping the arc length onto the lateral coordinate. This geometric mismatch increases the effective distance from the plaque center at off‐axis positions, resulting in marginally lower measured doses in the peripheral regions.

These findings demonstrate that the PFD system achieved an overall combined measurement uncertainty of approximately 4.3% (1*σ*), corresponding to an expanded uncertainty of about 8.6% (*k* = 2, 95% confidence level) when compared with manufacturer‐certified reference data. This represents an improvement over the conventional manufacturer method for plaque dose certification, which typically exhibits an expanded uncertainty of ±11% (*k* = 2). Both uncertainties were evaluated under the same expanded uncertainty criterion.

Previous studies on radiochromic film dosimetry, including EBT‐series films and flexible film dosimeters based on similar diacetylene polymerization mechanisms, have reported that total dose uncertainty remains relatively stable within the validated operational dose range.^18^, ^19^, ^23^, ^25^, ^38^, ^39^, ^40^, ^41^ The relatively stable uncertainty observed in Figure 9 across the investigated dose range is consistent with these reports, in which uncertainty variations with dose were generally small compared to the overall uncertainty level when measurements were performed within calibrated and clinically relevant ranges. Accordingly, Figure 9 is not intended to establish a general dose–uncertainty dependence but rather to demonstrate the operational stability of the proposed PFD system for Ru‐106 plaque QA.

The encapsulation layers were designed with asymmetric thicknesses to align the effective measurement depths of the films (1 and 3 mm) with the reference depths used in the manufacturer‐certified plaque dose data. This configuration ensured accurate depth‐specific comparison and consistency between measurements. The potential dosimetric impact of reversing the film orientation was estimated to be within 2%–3%, based on the reported Ru‐106 depth‐dose gradient, in which a 0.1 mm geometric difference corresponds to approximately a 2% dose variation.^44^ Therefore, maintaining a consistent film orientation during measurement is essential to ensure reproducibility and minimize directional bias in plaque QA applications.

The good agreement observed in this study can be partly attributed to the intrinsic material properties of the LiPCDA film. Chemically, LiPCDA shares the same diacetylene‐based polymerization mechanism as Gafchromic EBT‐series films (EBT3 and EBT4), which are well established as water‐equivalent radiochromic dosimeters. Because β‐particles emitted from Ru‐106 decay lose energy primarily through electron interactions in tissue‐equivalent media, the near‐water‐equivalent composition of the LiPCDA film provides comparable stopping‐power characteristics and thus a similar dosimetric response. Although previous LiPCDA studies have been limited to megavoltage photon irradiation, the present results demonstrate consistent behavior under β‐irradiation, supporting its applicability to low‐energy, electron‐dominated sources. This study therefore provides the first direct experimental validation of LiPCDA film behavior under β‐irradiation conditions, supporting the empirical transfer of photon‐based characterization data to Ru‐106 plaque applications.

These findings align well with earlier studies, such as Arjmand et al., who validated dose distributions for various Ru‐106 plaques using EBT3 film and Monte Carlo simulations.^13^ They reported gamma index passing rates >90% and dose profile agreements within 5%. Similarly, Eidi et al. evaluated three‐dimensional dose distributions for notched and circular Ru‐106 plaques using EBT3 film, diamond and diode detectors, and Monte Carlo simulations.^14^ Their results also showed excellent agreement with certified reference data. Our findings, based on a curved‐surface film dosimeter, align well with these results, offering improved conformity to plaque curvature, which marks a significant improvement over rigid dosimetry approach.

Film‐based dosimetry for Ru‐106 plaques has been shown to offer notable benefits while also presenting key challenges related to geometry and calibration. Several prior studies using EBT3 film and Monte Carlo methods revealed that spatial dose gradients and complex plaque geometries can introduce significant measurement uncertainties, underscoring the need for precise film alignment, calibration, and custom phantom setup.^11^, ^45^

In contrast, our system integrates a 3D‐printed QA tool and a dual‐layer film holder, coupled with the PFD, directly conforming to the concave plaque surface. This design effectively mitigates key challenges in curved‐surface dosimetry. It enables high‐resolution dose mapping with minimal geometric distortion and also eliminates angular discrepancies that commonly occur with flat film or chamber‐based systems. Collectively, these features show strong potential to overcome the inherent challenges of curved‐surface dosimetry.

The current PFD system was designed based on kirigami‐inspired structural principles, enabling the planar film to conform to the concave eye‐plaque geometry through controlled cuts and elastic deformation.^20^, ^21^ Specifically, two bi‐elliptical film segments were designed with radial or tangential kirigami‐inspired cuts along their periphery to enhance conformity to the dome‐shaped Ru‐106 plaque surface. This configuration was intended to improve contact stability by reducing wrinkling and detachment, thereby enhancing dosimetric accuracy.

Despite these efforts, the intrinsic geometric limitations of planar films constrain their ability to fully match the hemispherical curvature of eye plaques, especially at the peripheral regions. The customized dual‐layer film holder partially compensates for this mismatch by providing structural support; however, residual positioning uncertainties may still affect measurement precision. These limitations are consistent with previous studies demonstrating the inherent geometric mismatch and limited conformability of planar sheets to surfaces with nonzero Gaussian curvature.^36^ Given these limitations, a more accurate and robust solution may involve directly fabricating the film into a pre‐curved, hemispherical shape that intrinsically conforms to the plaque surface. Such an approach would eliminate the need for compensatory cuts or holders, potentially enabling more precise dose delivery verification, particularly for plaques with complex or asymmetric geometries.

In addition to the dosimetric agreement demonstrated in this study, the proposed plaque‐adaptive PFD system offers several practical advantages for clinical implementation. The customized QA tool was designed with a uniform thickness and a precisely machined curvature that conforms to the Ru‐106 eye plaque surface. This design allows reproducible and geometrically consistent positioning of the film. Consequently, the entire QA procedure can be performed in a single setup, eliminating the need for multiple detector placements and reducing setup‐related uncertainty.

A slight asymmetry in the film holder was intentionally introduced to ensure close conformity and stable contact between the plaque and the film, thereby minimizing geometric deviations on the highly curved surface. These features collectively provide clinically meaningful improvements in workflow efficiency and measurement reproducibility, even when the absolute accuracy is comparable to that of existing QA systems.

During prototype fabrication, curvature matching among the film, protective cap, and QA tool was identified as the most critical geometric tolerance. The final assembly incorporated precisely machined alignment grooves and surface markers to ensure reproducible positioning and consistent measurement depth across repeated setups.

In summary, the PFD system offers a practical, high‐resolution QA solution for Ru‐106 plaque brachytherapy. Its reliable alignment, strong agreement with certified dose data in both CAX and planar assessments, and ability to adapt to curved plaque geometries support its integration into plaque commissioning and routine QA workflows. Moreover, the PFD system could be adapted for basic research or for dosimetric characterization of other β‐emitting sources, such as P‐32 or Y‐90. This could be achieved by redesigning the holder geometry and cap structure to match the source curvature and dose distribution. Further development in film shaping and multi‐depth measurement will enhance its clinical applicability and robustness.

## 5 CONCLUSIONS

This study presents a PFD system optimized for commissioning and QA of Ru‐106 plaque brachytherapy. The system enables precise evaluation of both CAX depth doses and planar dose distributions on curved plaque surfaces. By employing a dual‐layer film holder and bi‐elliptical film shaping inspired by kirigami principles, the dosimeter achieved high spatial conformity to hemispherical geometries while ensuring reproducible measurements and optical stability.

CAX dose measurements at 1 and 3 mm depths agreed within ±0.4% of certified reference values across all plaque types, confirming the suitability of the system for verifying steep beta dose gradients. Planar dose distributions showed mean deviations within 3.3% in high‐dose regions, supporting its utility in assessing surface dose uniformity and detecting peripheral underdosage.

The inherent geometric limitations of planar films hinder full conformity to hemispherical plaque curvature, particularly at the periphery—a limitation that remains even with shaping strategies and is noted as an area for further improvement. In conclusion, the proposed dosimetry system offers a practical, high‐resolution, and reproducible solution for Ru‐106 plaque QA, enhancing the accuracy and safety of ocular brachytherapy.

## CONFLICT OF INTEREST STATEMENT

The authors have no relevant conflicts of interest to disclose.
